# Burnout Prevalence and Associated Factors Among Brazilian Medical Students

**DOI:** 10.2174/1745017901814010188

**Published:** 2018-08-31

**Authors:** Mariana Linhares Barbosa, Bárbara Lopes Rodrigues Ferreira, Thaís Nunes Vargas, George Martins Ney da Silva, Antonio Egidio Nardi, Sergio Machado, Leonardo Caixeta

**Affiliations:** 1 Unit of Pedagogical Support - Unievangélica Faculty of Medicine, Anápolis, Brazil; 2 Professor of Psychiatry – Unievangélica Faculty of Medicine, Anápolis, Brazil; 3 Laboratory of Panic and Respiration (LABPR), Institute of Psychiatry (IPUB), Federal University of Rio de Janeiro (UFRJ), Rio de Janeiro, Brazil; 4 Laboratory of Physical Activity Neuroscience, Physical Activity Sciences Postgraduate Program, Salgado de Oliveira University, Niterói, Brazil; 5 Associate Professor of Neurology, Internal Medicine Department, Faculty of Medicine, Federal University of Goiás, Goiânia, Brazil

**Keywords:** Burnout, Medical students, Mental health, Occupational health, Problem-based learning, Psychological responses

## Abstract

**Objectives:**

This study aims to identify the prevalence of burnout and associated factors in Brazilian medical students.

**Methods:**

In the largest medical school from Central Brazil, the Maslach Burnout Inventory-Student Survey and a socio-demographic questionnaire were adopted in this cross-sectional study. Correlations among the three dimensions of burnout were verified.

**Results:**

The evaluation of 399 students revealed a frequency of 12.0% of burnout. Women had a higher rate of burnout (8.0%) than men (4.0%). The fifth period had the higher frequency of burnout (27.1%), while the seventh grade had the lower frequency (2.1%). The students showed high scores only in emotional exhaustion (63.2%). Between the dimensions “emotional exhaustion” and “disbelief” found a significant correlation.

**Conclusion:**

The fifth period of the Medicine course and the female gender are the most affected by burnout syndrome and therefore, the preventive actions to reduce stress among medical students should be directed mainly at these higher risk categories. The low burnout rate found in our study can attest that the PBL methodology and medical schools strategically located to meet regional demand may represent strategies for the prevention of burnout among medical students.

## 1 INTRODUCTION

Psychological responses to stress including burnout represent an important issue in social neuroscience. Burnout is a syndrome constituted by the triad “emotional exhaustion”, “disbelief” and reduced “professional efficacy”. It is characterized as an inadequate response to chronic emotional stress; thus, it would be a way of adapting to difficult situations [1-7]. The most accepted definition today is based on the socio-psychological perspective, which considers the syndrome as a reaction to chronic emotional tension by constantly and excessively dealing with people, as occurs in the case of health professionals, educators and caregivers [6, 8, 9].

Some authors report that Burnout Syndrome affects 20 to 50% of health professionals [10]. It can also be described in individuals who perform student activities or professional preparation, subject to physical, emotional and intellectual wear similar to that of the worker, as is the case of medical students [6, 9].

The medical course is one of the most disputed among university selective processes, so the emotional impact of this choice comes even before graduation. However, many who choose to pursue a medical career do not have much knowledge about the routine experienced, both in the undergraduate and in the professional life itself [11, 12]. When these students begin medical graduation, they become continuously exposed to stressors throughout their training, due to factors such as excessive study load, little time for leisure and for the family, difficult choice of a specialty; stressors that may be worsened by one's own personality traits - obsessive traits, self-willed, perfectionism - which, if persistent can trigger the syndrome. Thus, due to the high emotional exhaustion and possible mental disorders that may occur during training, there are several investigations studying the syndrome in the medical courses [4, 6, 9]. These studies demonstrate the occurrence of stressful events during medical training, inducing “emotional exhaustion” and “disbelief” that may extend until after graduation, affecting the performance of the professional and his satisfaction with the work [4, 6].

Research carried out among undergraduates of the medical course revealed a prevalence of 10% to 45% of the syndrome, and in these studies, only two of their dimensions (“emotional exhaustion” and “disbelief”) were used as diagnostic criteria [6]. In the studies, that had a more rigid criterion, using the three dimensions, the values ​​were smaller. Costa *et al*. [6], for example, found a prevalence of 10.3% in a sample of 369 students (69.1% of the total students) using the three dimensions criterion. In a longitudinal study [4] that followed a class during six years of training, it was shown that among the three dimensions of the syndrome, the score for “professional effectiveness” increased during the course, accompanied by an equal elevation of the score for “disbelief”. The elevation of the score in the dimension “professional effectiveness” is a sign of emotional health, the opposite occurring in the syndrome. Already the elevation of the score in “disbelief” points to greater wear and tear along the course. “Emotional exhaustion” at the end of the course decreased, thus reducing the score on the scale used in this study. It was also verified that the third and fourth-year undergraduate students presented the most emotional disorders.

Due to exposure to stressors that would be harmful to the mental health of medical students since the beginning of their academic training, and that may disrupt their professional performance, more research is needed in other Brazilian regions to better characterize the problem, and to find variables that to assist in future and necessary prevention and treatment attitudes for the benefit of these students. Thus, this work aimed to investigate the frequency of Burnout Syndrome in academics from the first to eighth periods of the major medical course of Central Brazil, as well as to relate the Burnout level with the sociodemographic data of the sample studied.

## 2 METHODOLOGY

### 2.1 Research Design and Data Collection

The study design was a cross-sectional one. The research was conducted at the largest medical school in Central Brazil, at the Centro Universitário de Anápolis (Unievangélica), State of Goiás, with the population consisting of medical students from the first to the eighth period during the second semester of 2015.

The Medicine course offered by the Centro Universitário de Anápolis (Unievangélica) is based on the teaching methodology called Problem-Based Learning (PBL). This new methodology presents to the students a question related to health care or the clinical picture of a patient, from which, and after initial analysis, the students define their learning objectives and search the necessary sources for the information that answers the problem found and then report what they found and what they learned. Thus, it differs from the traditional teaching methodology, since it stimulates the student to be an active agent in his/her own learning process.

Students were informed about the characteristics of the research and the voluntary nature of participation. Students who did not wish to participate in the study and who were less than 17 years of age were excluded.

### 2.2 Sample Size

Sampling was of the non-probabilistic and convenience type, calculated according to the formula for simple (random) representative sampling, where “N” represents the student population, “n” sample size, and ” n_0_” the first approximation of the sample size: n = N. n_0_ / N + n_0_ [13, 14]. The formula used to calculate ” n_0_ ” is as follows: n_0_ = 1 / E_0_^2^. In this formula, E_0_^2^ represents the tolerable sample error, with value, adopted in this research, equal to 5% (0.05). Therefore, the value of n_0_ adopted in the present study was 400 [13, 14].

### 2.3 Instruments

Data collection was carried out through the Maslach Burnout Inventory-Student Survey (MBI-SS), a form adapted by Carlotto & Câmara [15], based on the version by Schaufeli *et al*. [16]. Data were also collected in a supplementary questionnaire the socio-demographic data to characterize the sample - age, gender, marital status (single, married or equivalent, divorced, widowed), home state, nationality, skin color. The Maslach Burnout Inventory-Student Survey (MBI-SS) consists of 15 questions, addressing the three dimensions of Burnout Syndrome: “emotional exhaustion (5 questions),” “disbelief (4 questions),” and “professional efficacy (6 questions).” All items are evaluated on a seven-point Likert scale, ranging from zero (never) to six (always) [15]. The inventory evaluates each dimension of the syndrome separately, according to the scores. High scores on “emotional exhaustion” and “disbelief” added to low scores for “professional effectiveness” indicate a high level of burnout. Studies on student burnout demonstrate that MBI-SS is an adequate and reliable scale [15]. Maroco & Tecedeiro [17] suggest as a cut-off point for the questionnaire translated and validated for the Portuguese language the following values: score above 14 for “emotional exhaustion”, above 6 for “disbelief”, and below 23 for “professional efficacy”. The scale is reversed in the dimension of “professional effectiveness”, that is, the higher the score, the more satisfied the respondent is and the less likely he/she will be to develop burnout [18].

### 2.4 Data Analysis

The collected data was tabulated in Microsoft Excel 2010, version 3.0, and analyzed using SPSS software for Windows, version 20.018. The level of significance was set at p <0.05. The following variables were analyzed: a period of the course, age, gender, skin color, marital status, home state and nationality, and burnout indexes (“emotional exhaustion”, “disbelief” and “professional efficacy”).

The Kolmogorov-Smirnov normality test was performed, which presented a normal distribution of the variables indicative of Burnout Syndrome. A descriptive statistic was carried out in the form of simple frequency and percentage. Pearson's Chi-square test was used to compare the percentage distribution. A Pearson Correlation was performed with age and quantitative values for the syndrome indicators, and in addition, a Simple Linear Regression (Stepwise) verified how each dimension of Burnout influences the others, comparing them to each other.

### 2.5 Ethical Aspects

The students who accepted to participate signed the Free and Informed Consent Term (IC). This research was approved by the Research Ethics Committee of the Centro Universitário de Anápolis (Unievangélica) (CAAE 40789214.7.0000.5076).

## 3 RESULTS

This study included 399 students from the first to eighth grade, number above the minimum calculated for representative sampling. The age of the sample ranged from 17 to 52 years, with a mean of 21 years (SD = 3.6). Of the participants, 222 (55.6%) were women and 177 men (44.3%). Most of the sample came from Goiás State (85.9%). Almost all the students were single (98.0%) and most considered themselves as white color (54.1%). Considering the total sample (399 students), 12.0% had a positive burnout index, of which 32 (8.0%) were women and 16 (4.0%) were men.

The results are presented in Tables **1** to **3**. Table **1** shows the frequency of positive burnout index, based on the criterion of the three dimensions (need to score for burnout in the three dimensions), in each period and according to sex. It was considered the cut-off points proposed by Maroco and Tecedeiro [17] for the Maslach Scale for Students (MBI-SS): Score above 14 in the dimension of “Emotional Exhaustion” (EE), above six in the dimension of “Disbelief” (DE), and below 23 in the third dimension, “Professional Efficacy” (PE). Table **2** estimates the positive frequency obtained in each dimension of burnout.

The verification of how much each dimension influences the others, by comparisons of all of them to each other, is presented in Table **3**. The dimensions EE, DE and PE in this Table are in numbers of students with “emotional exhaustion”, “disbelief” and low “professional effectiveness”, respectively. For the dimension “emotional exhaustion” it was obtained that the other two dimensions (“disbelief” and “professional effectiveness”) together are responsible for 26.2% of the positive cases, both with positive influence. In relation to the “disbelief” dimension, there is a negative influence of “professional effectiveness” and positive “emotional exhaustion”, totaling 36.1%. The dimensions “emotional exhaustion” (positive influence) and “disbelief” (negative influence) interfere in 15.1% of cases of low “professional efficacy”.

Through the correlation of Pearson, presented in Table **4**, it was possible to verify correlations between the three dimensions of burnout. Between the dimension “emotional exhaustion” and “disbelief” was found a significant correlation (r = 0.508; p = 0.001); Between “emotional exhaustion” and “professional efficacy”, there was a weak and negative correlation, that is, they were inversely proportional (r = -0.118; p = 0.018); The “disbelief” dimension also correlated negatively and moderately with “professional efficacy” dimension (r = -0.383; p = 0.001).

## 4 DISCUSSION

In our study, we have obtained 12.0% of the sample (n = 399) with a positive burnout syndrome index, using the three-dimensional criterion. This figure is considered a low rate of burnout. This value was very close to that found by Costa *et al*. [6] in his sample of 369 medical students from Northeastern Brazil, where he obtained a positive index of 10.3% using the same criteria. British authors also found low levels in British medical students [19]. On the other hand, the frequencies of positive burnout index in many studies are higher than those found in our sample. For instance, in an American study [5], using the same methodology as ours, authors found moderate and high levels of burnout (varying from 21% in the first-year class, until 43% in the third-year class). Chang *et al*. found 55% of medical students with high burnout rates using the same methodology as ours. In another American study, using less conservative criteria, authors defined as many as 45% of American medical students in a multicenter study as burnt out [20]. These differences can be attributed to differences in the methodology employed, cultural aspects and resilience factors in that particular culture, as well as the type of pedagogical management employed in each institution [21]. One of the factors that may contribute to the low burnout rates found in our study (compared to the high American rates, for example), may be the fact that most of our students come from nearby cities (85.9% are from Goiás) or even from Anápolis itself, that is, the students have an affective comfort generated by the proximity of their relatives. Medical schools strategically located to meet regional demand (avoiding the migration of young students away from their families) may represent a strategy for the prevention of burnout among medical students.

The students in the fifth period had the highest burnout rate compared to the other participating groups (27.1%). The fifth period also presented the highest proportion of positive emotional exhaustion index: 15.9%. Several studies point to the third year (or fifth period) as a period especially vulnerable to burnout [22, 23]. In a longitudinal study developed at a medical school in Sweden [22] with students from the first to sixth periods of the course, it was pointed out that the problem of this stage would not be related to the quantity of subjects to be seen, but to the increase of the preoccupations with the future and professional competence. The authors also observed that tension, exhaustion and symptoms of depression are risk factors for the onset of the syndrome in the fifth period of the course [22].

Some researchers also show that it is around the fifth period that one perceives a problematic in relation to the process of disenchantment about what had previously been idealized in the first period of the course, or even before initiating it, being this disappointment precipitated by the more intense contact with patients [23-25]. Other studies have pointed out that when students engage in more direct contact with patients in the fifth and sixth periods, they are affected by anxious symptoms, in some cases because of fear of making mistakes, such as giving wrong diagnoses. This is also due to the difficulty of communication among medical students, teachers and clinicians [26-28].

In the group of students surveyed, it is observed that the majority is female, a Brazilian trend of the last decade in medical schools and among new professionals registered in the Councils of Medicine [29]. Women also have a higher frequency of the syndrome compared to men. Despite the difference found between gender, this was not statistically significant (x^2^ = 2.521, p> 0.05). When assessing the dimensions of burnout syndrome individually, women also had a higher level of “emotional exhaustion” than men, a finding already seen in other studies. For instance, in the study by Paro *et al*. [30], which encompassed 22 Brazilian colleges, women scored more “emotional exhaustion” than men, but scored less on the criterion of “disbelief.” In the present study, women represented 58.3% of the sample with a positive score in “emotional exhaustion” and 58.4% with a positive score in “disbelief”, following in part the trend observed in that study [30].

Although some authors point out that the levels of “disbelief” increase during the course, the group considered in this study did not score above the value considered acceptable for this dimension of the syndrome, nor did it show an increase according to the passage of the periods, indicating that these students did not become insensitive or cynical and ironic in dealing with others. According to the literature, this behavior occurs, in particular, with professionals and internal medicine [21].

The university center in which the present research was carried out adopts a non-traditional methodology of teaching, called Problem-Based Learning (PBL). This is a method of active education that supposedly differs from traditional teaching by encouraging the student to develop self-seeking skills, making the teacher a facilitator of the whole process, and no longer the traditional owner of absolute truth. Gomes *et al*. [31], in a bibliographic review covering articles from 1998 to 2008, showed some advantages acquired by students submitted to the PBL teaching method in relation to the traditional method, namely: better interpersonal communication, better team performance, and better relationship with the patient. In theory, all this would contribute positively to better preparation of the future professional, reducing their anxiety in relation to what awaits them. Following this line of thought, in a survey carried out among medical graduates of a private college in Rio de Janeiro (n = 703), there was a greater score of “emotional exhaustion” and lower “professional efficacy” among students submitted to the traditional curriculum (67.4%), when compared to those under the PBL method (32.5%) [32]. The low burnout rate found in our study can attests that the PBL method may represent a strategy for the prevention of burnout among medical students.

In the sample studied, we obtained statistically significant correlations between the dimensions of burnout syndrome, according to Pearson's Correlation. Between the dimensions of “emotional exhaustion” and “disbelief” were found a significant and positive correlation; between “emotional exhaustion” and “professional efficacy”, was weak and negative; between “disbelief” and “professional efficacy” the correlation was moderate and negative. These findings may suggest a probable cause-effect relationship between “emotional exhaustion” and “disbelief.”

## CONCLUSION

The major findings of this cross-sectional study of medical students from Central Brazil were that about 12.0% of the interviewed medical students had burnout and that the fifth period of the Medicine course and the female gender are the most affected by burnout syndrome, therefore, the pedagogical and preventive actions to reduce stress among medical students should be directed mainly at these higher risk categories. Besides that, the low burnout rate found in our study can attest that the PBL methodology and medical schools strategically located to meet regional demand (avoiding the migration of young students away from their families) may represent strategies for the prevention of burnout among medical students.

## Figures and Tables

**Table 1:** Positive burnout indexes percentage in each period and according to gender, related to the total sample of medical students.

**Periods**	**Women with positive burnout indexes (%)^1^**	**Men with positive burnout indexes (%)^1^**	**Positive burnout indexes (%)^2^**
**1º**	5.00	1.67	8.30
**2º**	15.20	6.52	20.80
**3º**	3.70	3.70	8.30
**4º**	5.88	5.88	12.50
**5º**	15.50	6.89	27.10
**6º**	4.54	0.00	4.20
**7º**	1.96	0.00	2.10
**8º**	14.28	8.57	16.70

**Table 2:** Positive frequency of the three dimensions of burnout by period of Medical school.

**Variables**		**Positive for EE (%)^1^**	**Positive for DE (%)^2^**	**Positive for low PE (%)^3^**
**Periods**	1º	11.50	9.60	11.10
	2º	13.90	16.30	18.90
	3º	15.10	12.40	10.00
	4º	12.30	10.10	11.10
	5º	15.90	17.40	20.00
	6º	9.50	14.60	6.70
	7º	11.90	8.40	10.00
	8º	9.90	11.20	12.20
**Total**		63.20	44.60	22.60

^1^X^2^=13.986 (p=0.051) ^2^X^2^=27.437 (p=0.001) ^3^X^2^=14.650 (p=0.041) Legend: EE = Emotional Exhaustion; DE = Disbelief; PE = Professional Efficacy.

**Table 3:** Simple Linear Regression by Stepwise method to analyze the contribution of each dimension (EE, DE and PE) to each other.

**Regression**	**SEE**	**R^2^**	**p**
EE = 10.481 + 0.582(DE) + 0.101(PE)	1.56	0.262	0.05
DE = 8.36 – 0.34(PE) + 0.438(EE)	1.37	0.361	0.001
PE = 27.772 + 0.93(EE) – 0.419(DE)	0.76	0.151	0.001

Legend: SEE = Standard Error of Estimate; R2 = coefficient of determination; EE = Emotional Exhaustion; DE = Disbelief; PE = Professional Efficacy.

**Table 4:** Pearson's correlation among burnout domains (EE, DE and PE).

–	**EE**	**DE**	**PE**
**EE**	r = 1	r = 0.508	r = -0.118
–	–	p = 0.001	p = 0.018
**DE**	–	r = 1	r = -0.383
	–	–	p = 0.001
**PE**	–	–	r = 1

Legend: r= Pearson's correlation; EE = Emotional Exhaustion; DE = Disbelief; PE = Professional Efficacy.
